# Exploring the factors that contribute to the successful implementation of antimicrobial resistance interventions: a comparison of high-income and low-middle-income countries

**DOI:** 10.3389/fpubh.2023.1230848

**Published:** 2023-10-13

**Authors:** Tiscar Graells, Irene A. Lambraki, Melanie Cousins, Anaïs Léger, Patrik J. G. Henriksson, Max Troell, Carolee A. Carson, Elizabeth Jane Parmley, Shannon E. Majowicz, Didier Wernli, Peter Søgaard Jørgensen

**Affiliations:** ^1^Global Economic Dynamics and the Biosphere, Royal Swedish Academy of Sciences, Stockholm, Sweden; ^2^Stockholm Resilience Centre, Stockholm University, Stockholm, Sweden; ^3^School of Public Health Sciences, University of Waterloo, Waterloo, ON, Canada; ^4^Global Studies Institute, University of Geneva, Genève, Switzerland; ^5^Beijer Institute of Ecological Economics, Royal Swedish Academy of Sciences, Stockholm, Sweden; ^6^WorldFish, Penang, Malaysia; ^7^Centre for Foodborne, Environmental and Zoonotic Infectious Diseases; Public Health Agency of Canada, Guelph, ON, Canada; ^8^Department of Population Medicine, Ontario Veterinary College, University of Guelph, Guelph, ON, Canada

**Keywords:** antimicrobial resistance, antibiotic resistance, resilience, success factors, interventions, public health, global health, high and low-middle-income countries

## Abstract

**Introduction:**

Antimicrobial resistance (AMR) is a challenge to modern medicine. Interventions have been applied worldwide to tackle AMR, but these actions are often not reported to peers or published, leading to important knowledge gaps about what actions are being taken. Understanding factors that influence the implementation of AMR interventions and what factors are relevant in low-middle-income countries (LMICs) and high-income countries (HICs) were the key objectives of this exploratory study, with the aim to identifying which priorities these contexts need.

**Methods:**

A questionnaire was used to explore context, characteristics, and success factors or obstacles to intervention success based on participant input. The context was analyzed using the AMR-Intervene framework, and success factors and obstacles to intervention success were identified using thematic analysis.

**Results:**

Of the 77 interventions, 57 were implemented in HICs and 17 in LMICs. Interventions took place in the animal sector, followed by the human sector. Public organizations were mainly responsible for implementation and funding. Nine themes and 32 sub-themes emerged as important for intervention success. The themes most frequently reported were ‘behavior’, ‘capacity and resources’, ‘planning’, and ‘information’. Five sub-themes were key in all contexts (‘collaboration and coordination’, ‘implementation’, ‘assessment’, ‘governance’, and ‘awareness’), two were key in LMICs (‘funding and finances’ and ‘surveillance, antimicrobial susceptibility testing and preventive screening’), and five were key in HICs (‘mandatory’, ‘multiple profiles’, ‘personnel’, ‘management’, and ‘design’).

**Conclusion:**

LMIC sub-themes showed that funding and surveillance were still key issues for interventions, while important HIC sub-themes were more specific and detailed, including mandatory enforcement, multiple profiles, and personnel needed for good management and good design. While behavior is often underrated when implementing AMR interventions, capacity and resources are usually considered, and LMICs can benefit from sub-themes captured in HICs if tailored to their contexts. The factors identified can improve the design, planning, implementation, and evaluation of interventions.

## 1. Introduction

Antimicrobial ineffectiveness due to antimicrobial resistance (AMR) is a ‘One Health’ problem and social-ecological challenge that threatens sustainable development and public health (1–5). Considering the importance of antimicrobials in modern medicine, institutions and stakeholders have tried to address AMR and its consequences with interventions globally (6) as AMR contributes to higher healthcare costs (7, 8), and worse, to millions of deaths globally every year (9, 10).

Implemented AMR interventions have targeted many settings and scales with varying impacts due to the influence of the context in which they take place (11, 12). While interventions can enhance resilience toward AMR, information about AMR interventions and their social-ecological context remains limited (12). Bridging this gap may be key to building and strengthening resilience in human and animal health systems (6, 11, 13). There is a need to strengthen the design and implementation of AMR interventions with translatable information about their effectiveness. It is important to understand what key factors make interventions successful or hinder their success within and across a range of contexts that are still poorly known (14). Comprehensive frameworks, such as AMR-Intervene, aim to detail relevant information about both the interventions and the social-ecological context (11), but available information may be insufficient if there is incompleteness in intervention design or implementation, incompleteness or lack of reporting, or difficult and time-delayed assessments (12, 15).

Published interventions are a major source of knowledge in implementation science, but sometimes they do not follow established reporting guidelines, and if they do, these guidelines are insufficient for capturing relevant details of the social-ecological system (16, 17). Moreover, AMR interventions implemented in scarce resource settings, such as low-middle-income countries (LMICs), are not often reported publicly or published in scientific journals, whose publication fees challenge affordability in these settings (16). While studying the success of AMR interventions published in the scientific literature has provided promising insights for high-income countries (HICs) (16), there is a more limited understanding of the factors leading to success in LMICs—a knowledge gap that requires urgent attention and that our study aimed to address.

Although implementation science based on evidence takes time (12), exploring the context in which AMR interventions happen and what information can be obtained from the people who implement them may allow us to learn and enhance resilience toward AMR (16, 17). For that purpose, we used a questionnaire and thematic analysis to capture context and important factors contributing to intervention success, where success was briefly defined as the intended goal and what each intervention wanted to achieve (16, 18). This exploratory analysis aimed to compare factors for success in HICs and LMICs to help us understand whether there are themes related to success that may be universal and others that may be context-dependent. To our knowledge, this is the first study designed to identify AMR interventions implemented in LMIC and HIC contexts and the factors that contributed to positive outcomes in an effort to understand what factors need to be prioritized in each context.

## 2. Methods

A questionnaire was developed based on the AMR-Intervene framework to contextualize the social-ecological system (11, 12), and it included specific questions about success factors and obstacles to intervention success. The final questionnaire was designed using Qualtrics Online Surveys and consisted of 50 questions. Participants had the option to take the survey in English and Spanish. The time to complete the survey was approximately 30 min for each reported intervention.

We conducted a scan of potential participants who worked on AMR or in industries or settings that can be impacted by AMR (e.g., farming industries) and could be knowledgeable about AMR interventions. Potential participants were identified through: (1) our consortium network; (2) public sources such as the World Health Organization repository National Action Plans on AMR; (3) web-based searches; and (4) official websites of governments, industries, and non-governmental organizations. Potential participants were classified based on the regions defined by the World Health Organization (WHO; Africa, America, Eastern Mediterranean, Europe, Southeast Asia, and Western Pacific) and the potential sectors to ensure geographic and professional diversity. We used three different distribution methods: (1) potential participants identified were invited to participate in the study via email with a survey link, (2) distribution of the survey link through email via AMR networks such as ReACT, WorldFish, and STRAMA; and (3) survey available at the project website.^1^ Three reminders were sent via email and one through AMR networks, and the questionnaire was closed after 1 month of the survey’s implementation in June 2019. Participants who wanted to answer the survey had to be knowledgeable about interventions, were directed to an information letter that described the study, indicated our interest in preference reporting on interventions not published in the literature or reported elsewhere, although they were not restricted to this, and gave consent for their participation.

Interventions were analyzed in their social context (e.g., income, location, and agents responsible) and ecological context (e.g., microorganisms and level of resistance). The context of the social-ecological system was analyzed using the AMR-Intervene framework (11). For obstacles and intervention success factors, we performed an inductive thematic analysis to capture themes that contribute to positive outcomes from interventions tackling AMR, following the standard for reporting qualitative research (Supplementary Table S1) (18, 19). Inductive coding was performed using MAXQDA v.2020, a computer-assisted qualitative data analysis software, without a pre-existing coding frame, which allowed the data to drive themes. Two more co-authors (IAL and MC) independently coded a sub-set of 20 (26%) responses to assess inter-coder reliability and to limit bias from the main researcher, whose experience involves clinical microbiology and epidemiology. Coders had 90% agreement, and many different points of view were easily resolved via consensus. A theme was defined as the main idea or concept behind the participant’s answer and could be broken into more specific sub-themes, which were detailed factors related to the main theme. Interventions mentioning a particular theme or sub-theme were included and counted once, defining frequency as the number of interventions that reported a theme or sub-theme. Redundancies were included to not miss out on information, but if themes or sub-themes were in the same data item, they were only counted once. Factors seen as key components for positive outcomes were organized together (total frequency), but we also considered if they were reported as satisfactory or obstructive (partial frequency). Thematic analysis is described elsewhere (18) (Supplementary Table S2). We performed Fisher’s exact tests in R (version 4.1.1) to see if statistically there were differences in our categorical themes and important sub-themes between the expected and observed frequencies depending on HIC and LMIC context. We performed Fisher’s exact tests to see if there were statistical differences in themes and corresponding sub-themes between HICs and LMICs and to see if there were differences in reporting them as factors or obstacles to success.

## 3. Results

This exploratory study collected data from 77 interventions and their contexts (Table 1 and Supplementary Table S3). The economic scale in which interventions were embedded showed a predominance of HICs (*n* = 57), almost 25% of interventions were reported in LMICs (*n* = 17), one was implemented locally in two countries (one HIC and the other LMIC), and two interventions had a global scope. Interventions were located in America (3 countries), Europe (7 countries), South-East Asia (2 countries), Africa (14 countries), globally, and in the West-Pacific Region (1 country) (Supplementary Table S3). Canada (*n* = 35), Sweden (*n* = 10), and India (*n* = 10) were the countries with the largest number of interventions reported. The sector in which most interventions were implemented was the animal sector, followed by the human sector (Table 1). The oldest interventions date back to 1949 and 1985, both implementing mandatory prescriptions for antimicrobials in veterinary medicine in Finland and Québec (Canada), respectively. However, most interventions were recent (starting in 2015 or later (*n* = 43)) and without an end date (n = 56) (Supplementary Table S3). Time-bound interventions (*n* = 21) had a mode duration of 3 years and an average duration of 4 years.

**Table 1:** Basic background information extracted from reported interventions using the AMR-Intervene framework (11).

Group (11)	Variables (11)	Categories	*N* = 77	Percentage (%)
Social system	Economic scale	High-income countries	**57**	75%
		Low-middle-income countries	**17**	22%
		Global or both high- and low-middle-income countries	**3**	3%
	Sector scale	Animal sector only	**37**	48%
		Human sector only	**22**	29%
		Animal and food sectors	**7**	9%
		Human and animal sectors	**7**	9%
		Human, animal, food sectors, and environment sectors (‘OH’)	**3**	4%
		Environment or plant sectors only	**2**	3%
		Not specified	**1**	1%
Governance	Agents responsible	Public sector (government-owned)	**36**	47%
		Public and private sector	**21**	27%
		Public and academic sector	**8**	10%
		Private sector (private owned)	**7**	9%
		Academic sector (university/research/scientific sector)	**4**	5%
		Public, private, and academic	**1**	1%
	Level of funding	Public funding	**37**	48%
		Public and private funding	**12**	16%
		Private funding	**5**	7%
		Without funding	**21**	27%
		Not reported	**2**	3%
Trigger / goals	Trigger of the intervention	Pressure on AMR (high AMU)	**27**	35%
		State of AMR (increase of AMR)	**17**	22%
		Pressure and state of AMR (high AMU, increase of AMR)	**9**	12%
		Drivers of AMR	**8**	10%
		Impacts of AMR	**7**	9%
		Pressure and/or state of AMR and impacts of AMR	**5**	7%
		Not known	**4**	5%
	Main strategy	Conservation (reduce/improve AMU)	**20**	26%
		Conservation and surveillance and/or other	**17**	22%
		Surveillance	**12**	16%
		Conservation and containment (reduction of AMR spread) or IPC	**12**	16%
		Conservation or surveillance and other	**10**	13%
		Other	**6**	8%
	Level of implementation	National	**38**	49%
		Sub-national or Regional	**27**	35%
		Inter-regional (different countries in the same area)	**6**	8%
		Local	**4**	5%
		International (Global)	**2**	3%
Bio-ecological scale	Type of microorganism	Bacteria	**41**	53%
		No specific	**33**	43%
		Bacteria and Fungi	**3**	4%
Assessment	Assessment of the intervention	In progress	**39**	51%
		Not-evaluated	**21**	27%
		Evaluated	**17**	22%
	Subjective evaluation	Positive	**72**	94%
		Neutral/Not sure	**3**	4%
		No	**2**	3%

AMR, antimicrobial resistance; AMU, antimicrobial use; incl, includes; IPC, Infection prevention and control; OH, ‘One Health’.Bolded values indicate number of interventions among the total 77 interventions.

**Figure 1:**
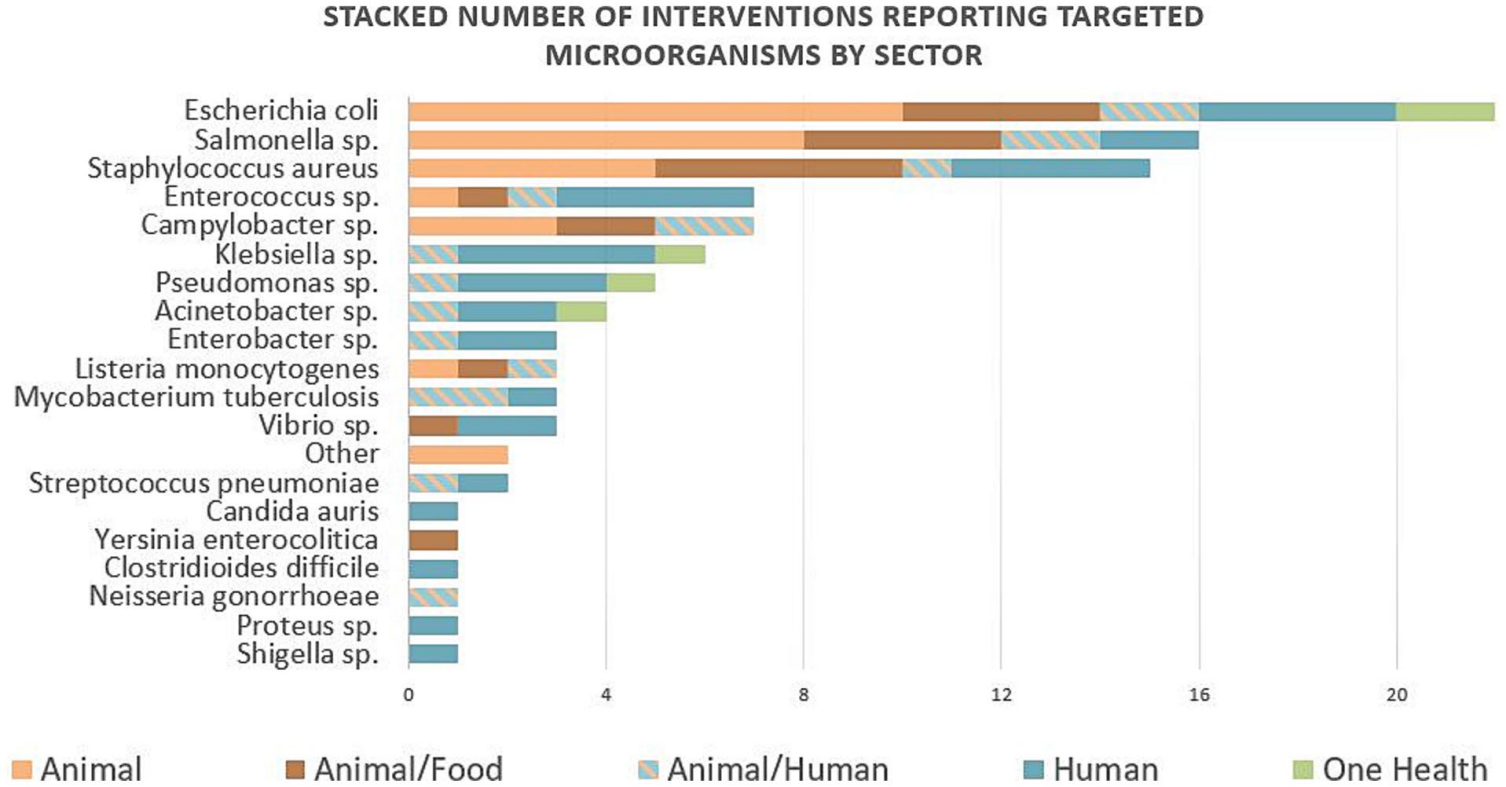
Number of interventions reporting targeted microorganisms stacked by sector. Twenty-seven interventions reported specific targeted microorganisms, and they were often targeting several microorganisms in the same intervention. The most reported microorganisms were *Escherichia coli*, followed by *Salmonella* spp., and *Staphylococcus aureus* targeted in 22, 16, and 15 interventions, respectively.

Looking at the governance system of the interventions, the governmental or public sector was responsible for and an actor in 66 interventions—alone or in co-responsibility with another sector (Table 1). Most interventions (70%) were funded, with the public sector being the major funder, while 27% of interventions (*n* = 21) had no specific funding source (Table 1). Interventions were triggered by high AMU (*n* = 27) or AMR prevalence (*n* = 17) or by their combination (*n* = 9). Thus, interventions were mostly reactive in response to a specific problem already happening (*n* = 71), while only a few were preventive (*n* = 6) (Supplementary Table S3). The main strategies used were to conserve the effectiveness of antimicrobials (e.g., reducing or improving AMU, 71%, *n* = 55) and surveillance of AMR and/or AMU (43%, *n* = 33). At the level of implementation, almost half of them were implemented nationally (*n* = 38) (Table 1). Almost 60% of interventions targeted bacteria, and one-third of interventions (*n* = 27) reported specific bacteria or the yeast *Candida auris* (Figure 1). The most reported resistance profile according to the standard definitions (20) at the start of interventions was multidrug resistance, which was present in one-third of the interventions (*n* = 25) (Supplementary Table S3).

Regarding intervention assessment, 21% were assessed, and of these, one-third reported positive outcomes—five reported decreased AMU and one decreased antibiotic resistance genes. Only three of the interventions assessed published their results in scientific journals. More than half of the interventions (51%) had the assessment in progress, and 27% did not consider the assessment when planning the intervention. However, subjectively, the majority of interventions were perceived to have positive outcomes. Overall, the cost-effectiveness of interventions was unassessed, but one intervention was highlighted as cost-effective. Another intervention (which was also perceived as non-successful) reported unintended consequences (outcomes that were not foreseen previously) that included annoyance and low self-esteem in some professional groups related to healthcare.

**Figure 2:**
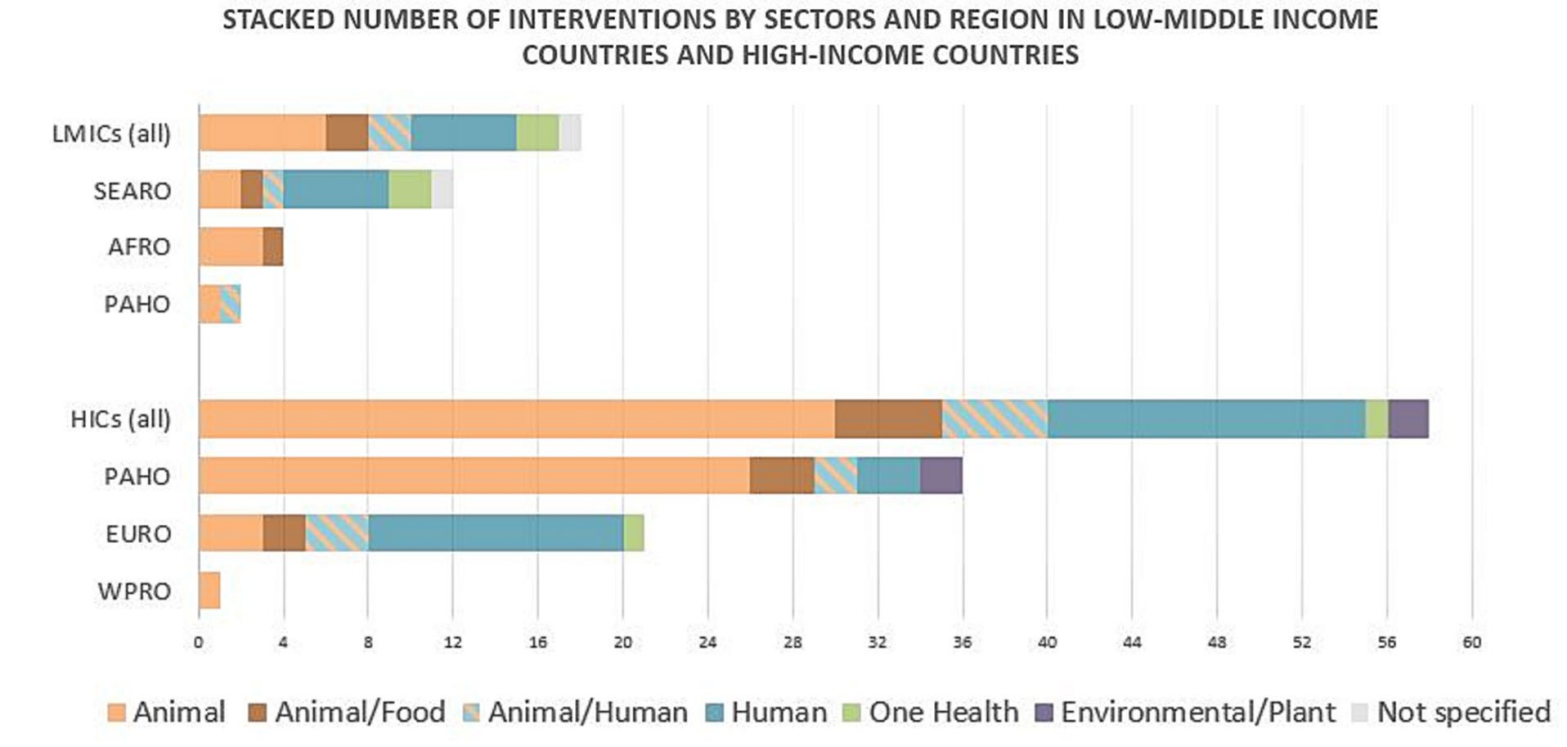
Stacked number of interventions by sectors and region targeted in low-middle-income countries and in high-income countries. Each group is disclosed per World Health Organization regions. AFRO, African region; EURO, European region; HICs, high-income countries; LMICs, low-middle-income countries; PAHO, Pan-Americas region; SEARO, South-East Asian region WPRO, West-Pacific region.

When comparing HIC and LMIC contexts, the timeline of interventions reported was similar in both groups, with most implementation done in the last 5 years. The agents responsible were, in most cases, public institutions. The animal sector was the most targeted in both LMICs and HICs, and the proportions of sectors were also similar between these two contexts, as shown in Figure 2. The triggers of most interventions were pressure on AMR with high or inadequate AMU and the increased state of AMR. Strategies used in both groups were the same and included four main groups or a combination of them: (1) conservation of antimicrobials with awareness or stewardship programs; (2) surveillance programs in AMR or AMU; (3) conservation of antimicrobials with regulations and policies to control AMU; and (4) infection prevention programs to control or contain AMR.

**Figure 3:**
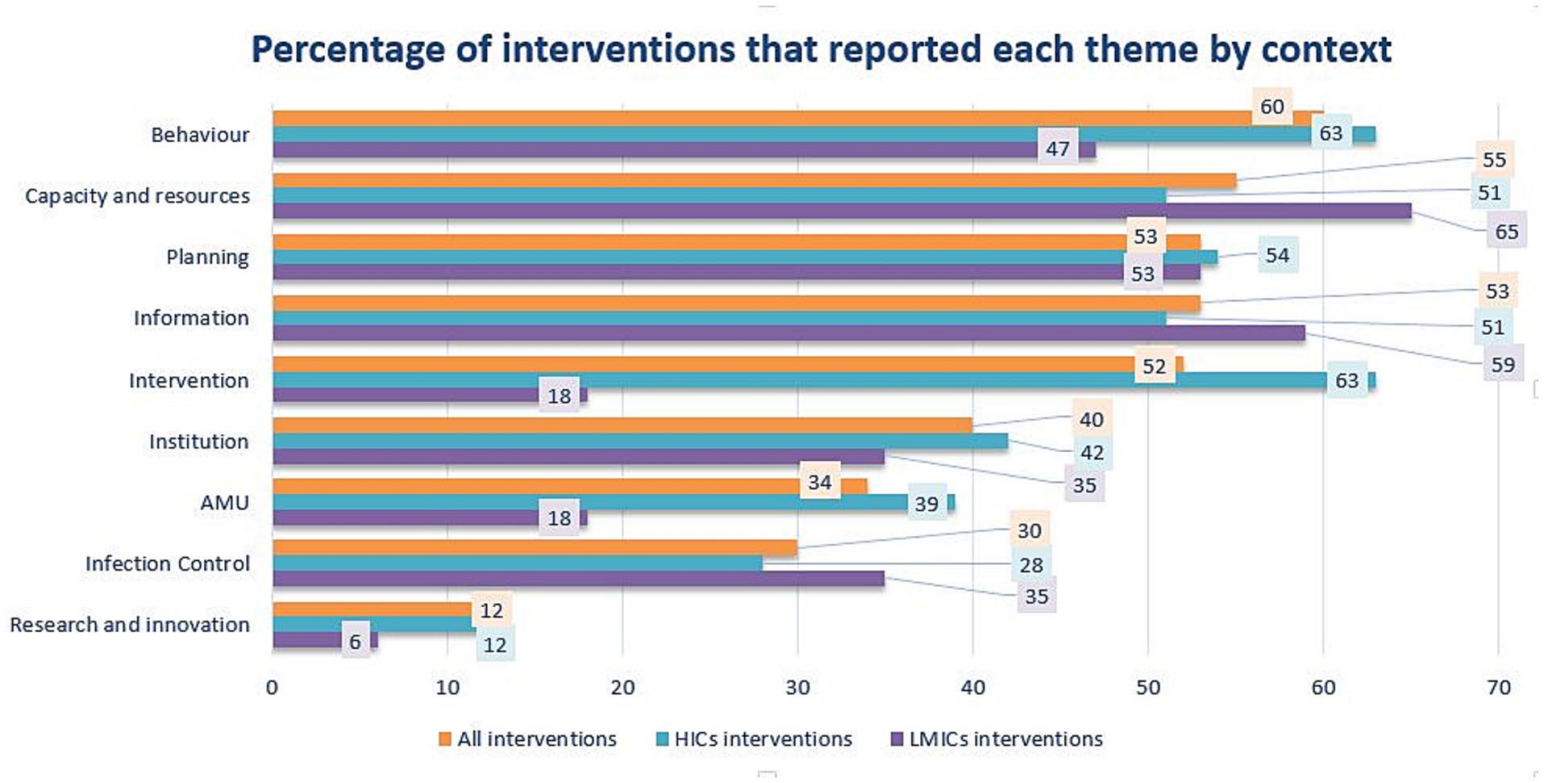
Percentage of interventions reporting each of the nine themes that were captured to lead toward positive outcomes of interventions. Percentage of themes for all interventions, high-income countries (HICs) and low-middle-income countries (LMICs) are represented in orange, blue, and purple, respectively. AMU, antimicrobial use.

Nine main themes and 32 sub-themes were captured in this exploratory thematic analysis (Figure 3 and Supplementary Table S4 (statistical results) and Supplementary Table S5). The most reported theme was behavior of individuals or institutions toward the intervention or its implementation, which included seven sub-themes that were: collaboration and coordination; commitment and engagement; trust and support; promoting, reinforcing, or awarding correct behavior; communication; frustration; and flexibility and adaptability. The second theme was the capacity and resources of the system where the intervention takes place and included three sub-themes, including personnel, funding, and premises and technology. The third theme was the planning of the intervention and included three sub-themes covering implementation, assessment, and design. The fourth theme was information available or resulting from the intervention, with five sub-themes including awareness, data availability, education, regulations/guides, previous experience or consultancy, and outcomes from the intervention. The fifth theme was intervention characteristics, which captured the qualities that make the intervention more prone to success and included four sub-themes: mandatory enforcement, multiple profiles, affordability, and preventive character. The sixth theme was institution features that influence the likelihood of positive outcomes, with two sub-themes: management and governance. The seventh theme was AMU, which captured the actions that affect use and had four sub-themes: access, reduction in use, improvement in use, and financial implications. Infection control was the eighth theme with two sub-themes: infection or AMR control; and surveillance, epidemiology, and preventive screening. The ninth, and last, theme was research, innovation, and novelty and included two sub-themes: new therapy and alternatives to antimicrobials; and investment.

**Figure 4:**
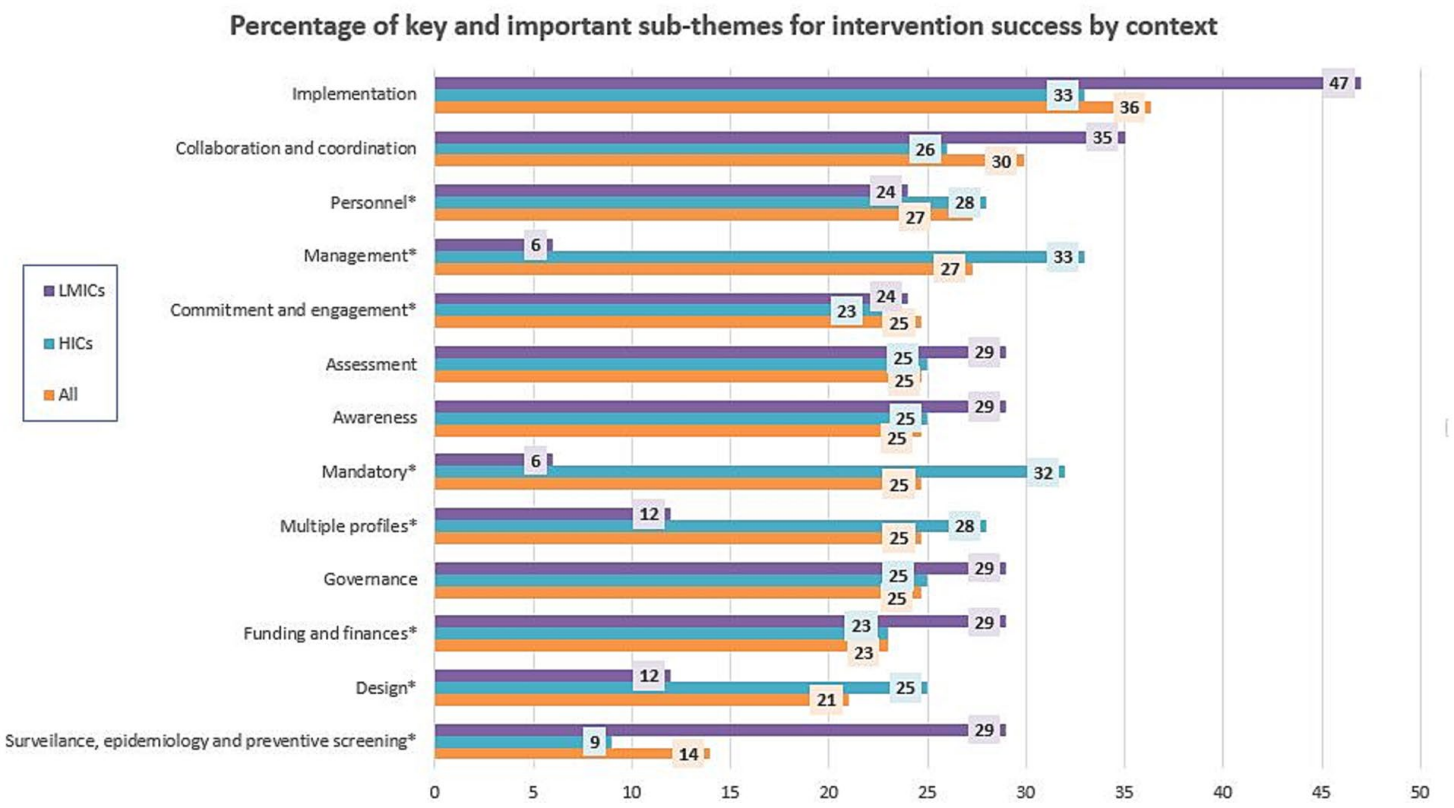
Percentage of sub-themes that were reported in at least one-quarter of interventions overall or by context. Key sub-themes are the ones not marked and important in all contexts, while sub-themes that were reported in a quarter of interventions for at least one of the groups are referred to as important sub-themes and are marked with (*).

The same sub-themes were reported in both HICs and LMICs except for the following five sub-themes that were reported only in HICs and not in LMICs: flexibility and adaptability; affordability; preventive character; financial implications inside the theme AMU; and investment in novelty and research. Most sub-themes enhancing or hindering the success of interventions were reported both as success factors and obstacles. Nonetheless, nine sub-themes were only reported as success factors (e.g., education), and one sub-theme (i.e., frustration) was reported just as an obstacle to intervention success (Supplementary Table S5). Ten sub-themes were reported in at least a quarter of all interventions, 10 also in a quarter of HICs, and seven sub-themes in a quarter of LMICs. Of those five, there were key sub-themes in all contexts. Eight were important sub-themes depending on the context and are detailed in Table 2 and Figure 4.

**Table 2:** Details of sub-themes reported in a quarter or more of interventions, HIC interventions, and LMIC interventions.

Theme	Sub-theme	Meaning and assumptions	Implications	Quotes
Behavior	Collaboration and coordination	Actors need to collaborate and/or coordinate themselves to enhance the likelihood of success. Collaboration and coordination lead to effective communication and implementation. On the contrary, reluctance to participate, lack, or difficult collaboration, disagreements, and lack of coordination with mixed tasks can jeopardize the intervention.	A collaborative and coordinative behavior is crucial to involve individuals in AMR and to engage them in the specific tasks they need to do with motivation.	“open collaboration between all entities”// “cooperation with food producers and cattle farms…”// “…and collaborative effort between industry and government”// “A key factor is the good collaboration between national and local […] Groups…”// “Challenges included attaining industry buy-in and collaboration, considering that each livestock industry has its unique considerations…”
Behavior	Commitment and engagement	Commitment, engagement, and implication of actors are crucial to conducting things well and in a positive way as people welcome the campaign and have the willingness to take actions including voluntary actions.	Actors who are committed believe that what they are doing helps in tackling AMR and are more aware and willing to (voluntarily) participate and take action.	“The swine stakeholder (…) voluntarily committed to reduce antimicrobial use by 20%” // “Implication of the stakeholders, communication with the staff” // “The initiative was also successful due to outreach and engagement with farm and veterinary communities …” // “Challenges included engagement and negotiation with industry around costs of antimicrobial stewardship,”
Capacity and resources	Personnel	Personnel and/or trained personnel working on the intervention. On the contrary, lack of them, or personnel with heavy overload schedules without sufficient time or personnel unable to assist, for example in remote or rural areas hinder intervention outcomes.	Personnel accessible, dedicated, and with enough time to carry out the intervention or only working on the intervention is needed to ensure the likelihood of success and that the actions expected from the interventions are met.	“availability of human resources” // “Personnel exclusively dedicated to that” // “availability of time, work initiated in the summer” // “Lack of experts and public diagnostic facilities for AMR-prevention”// “The availability to inf[ectious] dis[eases] specialists and the financing of the time it takes to do the rounds”
Capacity and resources	Funding and finances	Enough budget and funding to carry out all aspects needed for interventions over time. Funding for resources, techniques, or personnel, but also for teaching and training the main actors responsible for the intervention.	Good budgets are key as costs can be very expensive for implementing interventions. Without enough budget, many interventions are not going forward, are partially applied, interrupted, or side cost effects are assumed by others (with negative effects).	“Founder donor agency go through a complicated process which causes interruption of funding.” // “There was no dedicated budget for this campaign. Communication strategy was based primarily on the information relay and the ability of each organization to pay for the printing of the tools and their distribution.”// “Financial resources and education” // “Funding”
Planning	Implementation	Implementation planning needs to be very detailed, easy to apply, and considering the flexibility of contexts and to be tailored to them. It must also have consultation or guidance for actors during implementation to clarify the actions and objectives of the intervention. When lacking, often implies insecurity toward the intervention and actors can go back to old habits especially if the implementation process is long or requires a certain amount of time.	Strong implementation considers small-scale contexts (e.g., regional) even though interventions can be implemented at bigger scales (e.g., national). Guidance enhances positive outcomes, even though the implementation is a long, process as they can rely on experts or other professional’s criteria when doubts arise. It promotes the self-esteem and motivation of executors due to continuous knowledge, feedback, and follow-ups.	“…there are provincial and regional production differences, so a national requirement has to be flexible enough to take these differences into account…”// “It takes a lot of time to implement a program that is supposed to reach all nurses in all hospitals”// “resistance to change - this change took over 10 years to implement!”// “But we also understood that it would take time to implement in all hospitals…Step by step we learn more with national and regional workshops to share experience”// “[Implementation] Guidance from WHO, OIE and FAO” // “Support from WHO & AGISAR documents”
Planning	Assessment	After implementation, checking, analyzing, or measuring outcomes of the actions applied can help to elucidate the usefulness of the intervention or its possible gaps, otherwise, the usefulness is not assessed and, therefore, unknown.	Results from the assessment can help to maintain motivation if there are positive outcomes and to identify new goals and opportunities to improve outcomes or to promote actions impacting AMR.	“Dialogue based on the figures for each unit and they can see differences between units and colleagues.” // “[Assessment with] quantifiable objective”// “Clinical microbiology laboratories are typically required to provide annual [data]… to providers to help guide empiric antimicrobial therapy.”// “to obtain enough microbiological data to follow temporal trends of antimicrobial resistance.” // “Impact of awareness creation needs to be evaluated”
Planning	Design	Time to plan and design interventions. Good planning has well-defined targets and detailed timeline, and it can foresee if training of professionals is needed or if possible complications and where can arise.	Preparedness and time to carefully think about the system interventions are embedded is key to having the desired outcomes.	“This was discussed at length before the intervention was…implemented.” // “… systematic collection, aggregation and analysis of AMR data representing all the geographical areas of the country is being done. Lab capacity in terms of manpower development (through training by ASM members), providing laboratory SOP logistics and equipment has been developed, a software capable of collecting all the lab and epi[demiological] data has been developed to collect both kind of data from different sentinel sites to the center… in real time.”// “short timeline to create and deliver a national awareness campaign”// “Cost is always an obstacle as interventions typically add cost to operations; this is typically discussed before the intervention is finalized and implemented.”
Information	Awareness	Knowledge about AMR and people aware of the problem of untreatable infections enhance positive outcomes and thorough follow of therapy. Ignorance of the problem may lead to pressure for antibiotic prescribing and public opposition.	Society may behave differently following and finishing prescribed antimicrobial treatments. Prescribers are less pressured to prescribe treatments to please patients or farmers. Citizenship is engaged to preserve antimicrobial effectiveness.	“Consistent awareness creation, commitment” // “Public awareness by showing who received certificates. Certificates were handed over by publicly important personalities, such as health ministers or regional governors. - Public awareness through modern media (TV, radio)”// “This Annual Conference recalls the importance of the issue of antimicrobial resistance.” // “industry-wide initiative that expanded past our sector, and was accompanied by regular communication to farmers to increase awareness”
Intervention	Mandatory enforcement	When interventions are mandatory, actors need to implement and comply with what is mandated, independently of what they think or their preferences.	Intervention has to be implemented by the main actors, and they do not necessarily need to be interested (so it is not siloed to the ones who already care like voluntary interventions).	“Strict government regulation and requirement to reduce antimicrobial use in food animals at the national level”// “It was a mandatory reduction in use”// “it was successful because it was mandated - farmers had to comply”// “Regulatory authority saw it important to make sure that the legislation was obeyed.”
Intervention	Multiple profiles	Interventions whose responsible actors are from different sectors (multisector, One Health), disciplines (multidisciplinary/ transdisciplinary), or have different roles in the same action or complemented actions (multifaceted). Intervention is composed or carried out by different actors in sectors, settings, disciplines, or professional backgrounds.	Different professionals, sectors, and disciplines help to understand and detail better the variety and complexity of AMR and have more insights on how to tackle this challenge. Joined efforts from different backgrounds and perspectives may have bigger impacts and redundancies.	“…collaborative effort between industry and government” // “The cross border aspect, transdisciplinary, regional network formin[g]a common goal.” // “multi-disciplinary team from industry, academia and government” // “inability to accept multidisciplinary or varied thought processes”// “Multisector approach” // “One Health approach”
Institution	Management	Execution of interventions suggesting how interventions are going to be done (either designed, implemented, or assessed). Management has communication as a key skill to drive and organize all the pieces of the intervention.	Good management foresees how to train, how to coordinate, or how to enhance the collaboration of actors. This empowers and increases the information available in the system, plus it increases knowledge and self-esteem.	“That so many […] national agencies work together with the same problem and message to the public.” // “Regional training activities” // “Implication of the stakeholders, communication with the staff” // “open collaboration between all entities”// “Educational afternoons for them, updates and workshops. Practical information on how to treat infections.”
Institution	Governance	Compromise, commitment, engagement, support, and clarity toward the intervention, its goals, and decisions from the institution suggesting what should be done or being accountable for interventions.	Ensures balanced effort and the broader interests of the institution to maintain or to carry out the intervention. This is done, independently of individualities and personal interests, joining efforts in partnerships and avoiding hierarchy or roles of power.	“Long-term government engagement of stakeholders”// “Good political support” // “Governance of the programme and financial commitment” // “there is a need to strength[en] the relation between Academia-Governmental institutions”// “Achieve good multisector collaboration and bureaucratic procedures between institutions from different origins.”
Infection Control	Surveillance, epidemiology, and preventive screening	Information about the current epidemiological situation with continued surveillance, antimicrobial susceptibility testing, in some settings, preventive screening.	These tools can help to better manage AMR, and useful detailed data to know what is more prevalent including species and subspecies data.	“Understand epidemiology at a subspecies level, as species level does not allow to understand real epidemiology” // “- Implementation of preventive screening”// “Antibiogram development (i.e., antimicrobial resistance surveillance in human pathogens) has been common practice in clinical microbiology laboratories for many years.”// “…obtain enough microbiological data to follow temporal trends of antimicrobial resistance.”

AMR, antimicrobial resistance and AMU, antimicrobial use.

Fisher’s exact test to see if themes and sub-themes were reported independently of the HICs and LMICs context resulted in a value of p of 0.38 for all ‘themes’ and 0.18 for the most important ‘sub-themes’. If we look at how themes were reported, Fisher’s exact test for all themes showed statistical significance: for success factors of themes, the *p-*value was as low as 0.0005, and the test for obstacles had a *p*-value of 0.043. About important sub-themes, only the test for reporting success factors had statistical significance (*p*-value = 0.043), but not the test for obstacle reporting (*p*-value = 0.11). None of the Fisher’s exact test *p*-values for each theme and expected sub-themes were statistically significant (*p* > 0.05), and they are reported in Supplementary Table S4.

## 4. Discussion

Historically, tackling AMR has been dominated by strategies aimed at finding new antimicrobials and reducing the need for antimicrobials. However, the weak pipeline of research and lagging efforts for new antimicrobial drugs (21) have left the latter as almost the only option for addressing this problem, and that is how many institutions intervene in attempting to reduce or improve AMU or its drivers (e.g., stewardship programs, hygiene, or vaccines). This became clear from this exploratory study.

AMR responses targeting AMU rely on behavioral change to improving how we use antimicrobials and, if possible, reduce demand in multiple settings and at multiple scales. Therefore, it is fair that ‘behavior’ stood out as the most reported theme in this study, a finding that aligns with our previous study on HICs (16). Because many actors involved lack previous experience (22), effective interventions need policy enforcement because information alone, vague, or loose policies do not translate to the changes intended by governments or the healthcare system (23, 24). There is a need to target individual behavior and personal responsibility as AMR interventions are strongly influenced by personal attitudes and, in consequence, the behavioral choices made, which is known as the ‘ABC’ paradigm for social change (25). Contrary to other public health practices, such as tobacco or wearing a mask, the use of social norms is limited as the behavior of using antibiotics or other antimicrobials is not visible (26). Promoting a good environment and relationships between individuals and institutions (‘collaboration and coordination’) was key to enhancing positive attitudes. In our results, having good ‘governance’ for making (the right) decisions was also identified as vital for intervention success. The sub-theme ‘commitment and engagement’ of both individuals and institutions also emerged as an important success factor as it reflects the “arms” of interventions and good ‘management’ that enable actors to take effective action.

Predictably, the ‘capacity and resources’ of the system were the second key theme. ‘Funding and finances’ were identified as vital for the success of interventions, and worrisome is that (lack of) ‘personnel’ has been highlighted as an important sub-theme hampering chances of success due to shortages, time overloads, and untrained actors that can lead to risky behaviors and actions contributing to the spread of AMR (22, 27). In this line, public ‘awareness’ may help to increase budgets for addressing AMR (and, therefore, increase the ‘capacity and resources’ of the system and for hiring personnel whose executive role is crucial).

Probabilities of positive outcomes in AMR interventions also rely on careful ‘planning’, which was the third theme in importance, with good ‘implementation’, ‘assessment’, and ‘design’ sub-themes being key. Good planning considers the capacity of the system and resources available at the time of implementation, but it should also detail how behavioral change is impacted. The description must include which actors shall be involved; social, historical, economic, or political contextual factors that influence the behavior of interest; and the time and frequency needed to routinely accept and adapt the intervention to avoid the tendency to return to old habits (12, 28–30). It is obvious that the implementation of an intervention needs to be evaluated to determine its effectiveness, but we found that such assessments were often overlooked. We have a strong need for the results of interventions to understand whether and how interventions work, for whom, and under what circumstances. With this information, we could make adjustments to the intervention throughout the implementation process. Moreover, when designing interventions, it is important to consider that mandatory policy enforcement actions are often perceived as more effective, as they are not siloed to those already interested and everyone needs to comply. That effect could be seen with mandatory public health interventions in response to COVID-19, which were important contributors to decreased mortality, attenuated economic impact, or increased vaccination rates among young people with low-risk perception and had a long-lasting results before and after implementation (31, 32). However, people designing interventions need to be pragmatic and fully aware of their possibilities, infrastructures, and systems to not collapse those affected by interventions. Interventions that include ‘multiple profiles’ of experience were perceived to increase impact because more insights and broad knowledge were considered. In this sense, Canada was the country where implemented interventions accounted for many alliances between the public, private, and/or academic sectors to fight against AMR, which is encouraging.

Taking all themes together, we could see statistical differences in theme reports both in success factors and obstacles, meaning that there were differences in how they were reported. If we look at how the most important sub-themes were reported, only the reporting of success factors showed statistical differences between HIC and LMIC contexts. Statistical differences between each theme (and sub-themes) captured between HIC and LMIC contexts were not found. LMICs had ‘funding and finances’ and ‘surveillance, epidemiology, and preventive screening’ as key themes for success and did not have much detail about the issue other than that they are at early-stage phases that manifest that they are still developing these surveillance and epidemiological systems, while HICs had more factors of success that were more descriptive, specific, and detailed, such as ‘multiple profiles’, ‘mandatory enforcement’, ‘management’, or ‘design’. Therefore, themes and sub-themes leading to success seem to be similar independent on the context, but how they are perceived is different. LMICs would benefit from considering the sub-themes captured in HICs when developing AMR interventions for their contexts once they fulfill their basic needs for better resources, surveillance, and epidemiology.

Most interventions reported in this study were part of the ‘gray’ literature, meaning they were not published in scientific journals. The context of both HIC and LMIC interventions was studied with the AMR-Intervene framework (11). The broad context of interventions is often not considered in our simplistic interpretations of knowledge-driven practices (27), but from our analysis, we could see that most interventions were recent (from 2015 and later; e.g., only two interventions were implemented long ago) and implemented and funded by public institutions, such as governments and public alliances, independently of the context (e.g., tripartite (FAO-OIE-WHO) and interventions in the African region). These characteristics and timelines align with the triggers of interventions being reactive, intervening when a concern has already arisen rather than being preventive (Table 1).

Reported interventions in our study were triggered by the state of AMR, or AMU, which is commonly recognized as a major driver of AMR and is accelerated by misuse and massive use (33). Interventions targeted mainly AMU, and the main strategy of interventions focused on AMU reduction or improvement via awareness or antimicrobial stewardship programs; AMU surveillance; or AMU policies/regulations within the animal sector. In this study, the types of interventions implemented to addressing AMR were the same in LMICs and HICs. As an exception to that, only HICs reported interventions whose main strategy was infection prevention with the aim to address AMR at the upstream point.

Interventions in this analysis were implemented mainly in the animal sector (Table 1). In contrast, interventions published in the scientific literature focus predominately on humans historically (16, 17). This fact could be related to the diffusion of the survey, as in our scan there were many professionals working with animals. This is interesting as AMR information is difficult to access and may be delayed or unavailable to other peers in settings that are less engaged with publications, research, or academia, or in sectors that do not belong to human health but that are involved in AMR (e.g., dairy farms). Although interventions still target only one sector, the predominance of the animal sector in this exploratory analysis is inspiring because it makes visible the wide variety of interventions that are implemented outside human medicine, especially those related to animals, which also have an important burden in AMU (34).

Targeted microorganisms are important in human, animal, food, and environmental systems, which emphasized the importance of multisectoral approaches and the need to tackle AMR from a ‘One Health’ perspective (2). Many zoonotic diseases are related to food (e.g., *Escherichia coli*, *Salmonella* sp., *Staphylococcus aureus*, *Enterococcus* sp., *Campylobacter* sp., or *Klebsiella* sp.), which can hamper global progress toward the Sustainable Development Goals that have a direct or indirect relationship with AMR (35).

Wrapping up, key sub-themes reported in LMICs were focused on ‘funding and finances’ and ‘surveillance, epidemiology and preventive screening’. The countries belonging to the LMIC group in this study are included in the lower-middle-income group (36) and are mostly in early-stage phases of AMR interventions with a focus on developing their institutional infrastructures for improving epidemiology and AMR surveillance. Detailed information and specific comments that HIC interventions reported could be a good step for them. HICs often have better and well-established infrastructures and systems for surveillance and epidemiology, which allows for more awareness about details that are important to positive outcomes. In conclusion, LMICs would also benefit from carefully considering ‘multiple profiles’, ‘mandatory enforcement’, ‘management’, or ‘design’ reported in HICs as important factors when implementing AMR interventions if they tailor them to their particular contexts.

Conclusively, exploring success factors and obstacles separately is important for recognizing features that help interventions be effective but also features that can go unnoticed when they work or are taken for granted. Relying on only one theme (or sub-theme) will likely not have enough leverage to address AMR. However, combining them may positively impact reducing AMR, emphasizing the use of several approaches to maximize success. The complexity of the problem demands wider approaches involving ecological and biological, as well as social and psychological sciences (23, 37) because there are other components that select for AMR (38) or internal dynamics that can affect behavioral change and awareness (11, 12, 29). Applying a social-ecological lens will provide richer insights and a deeper understanding of factors affecting AMR and infectious diseases. Narrowing current knowledge gaps in this area may be possible by also including qualitative or mixed analysis to strengthen implementation science (28, 39, 40).

## 5. Strengths

The main advantage of this analysis is that it compares factors contributing to the success of interventions according to the socio-economic context in which they take place: HICs and LMICs. It also aimed to involve a wide audience that is engaged in AMR mitigation, either directly or indirectly, even though we cannot be sure about how successful we have been. Non-traditional stakeholders are needed (but often not considered) in addition to traditional stakeholders to identify multi-pronged and sustainable perspectives to tackle and reduce AMR and its impacts on humans, animals, and the environment (41). This exploratory analysis has generated information mainly from non-published interventions, highlighting data that may have been overlooked to date. Interventions have been characterized in their social-ecological context, and the personal experience of those involved has made valuable information accessible to other colleagues independently of assessment. Broad system integration of health system components and the AMR-Intervene socio-ecological factors have been considered to study interventions that have been shown to positively enhance resilience and reduce knowledge gaps (42). To complete our study, we used thematic analysis, which is a flexible and consistent qualitative framework for capturing perspectives before evidence is available and for producing reports suited to inform policy development (18).

## 6. Limitations

Our goal of studying an approximately similar number of human, animal, and environmental interventions evenly located in the different WHO regions was not met, even though participants from organizations worldwide were invited to participate. Of all WHO regions, we were not able to engage the Eastern Mediterranean Region. Important themes may be missing for this region, because not all sectors and types of interventions reported were equally represented. Important themes in LMICs may be missing as contexts can be highly heterogeneous compared to HICs, which have better integration and organization in their health and surveillance systems (43–45). Even if countries are in the same income group, they may have different systems and regulations, and cultural, political, societal, or local circumstances that impact interventions, and while our survey covered a wide variety of these aspects, our study may not have sufficiently captured relevant details to AMR. Nevertheless, our exploratory study aimed to reach the broadest possible understanding of AMR interventions using the AMR-Intervene framework (11, 12) and what factors contribute to successful outcomes. The last limitation is related to the consequences of applying the identified themes to complex adaptive systems, as they can have different interactions that can cause outcomes that we cannot foresee. However, consistent reporting/monitoring, preparedness, and broad system thinking before implementing interventions are tools to anticipate and address unintended outcomes.

## 7. Conclusion

Perceived factors that are cornerstones for interventions to be successful were grouped into 9 themes and 32 sub-themes. To our knowledge, this exploratory approach is the first one aiming to engage a wide variety of stakeholders worldwide to cast light on factors that contribute to the success of interventions from different perspectives. Using this inclusive view and by applying a social-ecological lens, five key sub-themes emerged as universal in HICs and LMICs, while other sub-themes emphasized what must be considered differently in each. By capturing the experiences of interventions implemented in HICs whose basic needs and resources were covered, this study has helped to identify more detailed key factors for successful interventions. These identified factors can help strengthen policies and AMR intervention planning in LMICs as they can be applied and tailored to these resource-scarce settings. Building resilience toward AMR requires proactive approaches and novel insights from qualitative and behavioral sciences that are able to capture the heterogeneity and details that affect AMR.

## Data availability statement

The raw data supporting the conclusions of this article will be made available by the authors, without undue reservation.

## Ethics statement

The questionnaire followed the approved procedures by the University of Waterloo Research Ethics Committee and it was reviewed and received ethics clearance through a University of Waterloo Research Ethics Committee (ORE#40519).

## Author contributions

TG, IL, and MC: data collection and curation. TG: formal analysis. SM, DW, and PJ: funding acquisition. TG: methodology and writing—original draft. TG, IL, MC, AL, PH, MT, CC, EP, SM, DW, and PJ: writing—reviewing and editing. All authors contributed to the article and approved the submitted version.

## Footnotes

^1^ https://amr-resilience.gtglab.net
