# Supplementary material for: Exploring the factors that contribute to the successful implementation of antimicrobial resistance interventions: a comparison of high-income and low-middle-income countries
Source: Front Public Health. 2023 Oct 13;11:1230848. doi: 10.3389/fpubh.2023.1230848 (PMC10612146; doi:10.3389/fpubh.2023.1230848)
Supplement: Supplementary file 1 [file Data_Sheet_1.docx]

**Table S1**: Standard Reporting for Qualitative research (SRQR) (1).

| Number | Topic | Manuscript | Page |
| --- | --- | --- | --- |
| S1 | Title | x | 1 |
| S2 | Abstract | x | 2 |
| S3 | Problem formulation | x | 3 |
| S4 | Purpose or research question | x | 3 |
| S5 | Qualitative approach and research paradigm | x | 3-4 |
| S6 | Researcher characteristics and reflexivity | x | 4 |
| S7 | Context | x | 5 |
| S8 | Sampling strategy | x | 3-4 |
| S9 | Ethical issues pertaining to human subjects | x | 4 |
| S10 | Data collection methods | x | 3-4 |
| S11 | Data collection instruments and technology | x | 3-4 |
| S12 | Units of study | x | 4 |
| S13 | Data processing | x | 4 |
| S14 | Data analysis | x | 4 |
| S15 | Techniques to enhance trustworthiness | x | 4 |
| S16 | Synthesis and interpretation | x | 6-8 |
| S17 | Links to empirical data | x | 11 |
| S18 | Integration with prior work | x | 6-8 |
| S19 | Limitations | x | 9-10 |
| S20 | Conflict of Interest | x | 10 |
| S21 | Funding | x | 10 |

**Table S2:** Thematic analysis iterative process following the methodology described elsewhere (2)**,** reference number 17 in the main manuscript, and adapted to our study about obstacles and success factors in AMR interventions.

| 1. Familiarization |
| --- |
| Reading and reviewing answers several times, noting down details. |
| 1. Initial Coding |
| Bottom-up coding: detailed and specific ideas extracted from the answers. Coding was performed using MAXQDA v.2020, a computer assisted qualitative data analysis software, without a pre-existing coding frame and, therefore, was inductive, allowing the data to drive the themes (data-driven). Re-occurrences, peculiarities grouped in different codes. Very detailed codes. |
| 1. Identify potential themes |
| Inductive coding and generate more general themes to the differently ways of answering. To clearly conceptualize the research question and to prevent bias from the main researcher whose experience involves clinical microbiology and epidemiology, two co-authors independently coded responses to assess interpersonal consistency. The same text could be related to different codes if more than one idea was underlying. In general, there was agreement between ideas and discrepancies were discussed to assess slightly different points of view until final consensus. At this point, classification of themes was decided to be done in 3-levels: the highest level was defined as the theme or main topic, inside this one a second optional level of categories, and, the lowest level was defined as sub-themes. The third level or sub-theme was the most detailed to not miss any idea but could be clustered in other sub-theme afterwards. A theme was defined as the main explicit and clear tacit idea behind the participant answer and could be broken into sub-themes which were detailed specific factors related to the main theme. A category was a (optional) middle classification where sub-themes could be related in a smaller group. First themes extracted from answers. |
| 1. Reviewing themes |
| Reviewing codes in phase 3. Requirements for extracting general patterns often need a certain amount of data and repetition. Otherwise, they were anecdotal and subsequently excluded. Frequency was counted as interventions mentioning a particular theme, category, or sub-theme independently of the number of times repeated on each data item. Redundancies were included to not miss information, but if present in the same data item they were counted as one. Clusterization of codes (themes and subthemes) with similar or equal connotations. |
| 1. Defining themes |
| Defining key themes and sub-themes that had similar or the same meaning but different perspectives. Refining of themes. Factors seen as key components for positive outcomes (either because factors were seen satisfactory or the opposite, or lack of, obstructive) were re-organized and clustered together (total frequency) although each contribution was considered (partial frequency). Integration and meaning with the conducted research question: causes for success of interventions tackling antimicrobial resistance, and differences between the two groups studied according to countries income. |
| 1. Thematic analysis report |
| Outcomes of the captured themes when addressing AMR: meaning, assumptions and implications of each theme, report most mentioned themes (frequency) and sub-themes. Graph comparing both groups studied. Qualitative relation between sub-themes were mapped using the online software draw.io and sub-themes were connected when a respondent overlapped or implied tacitly a certain overlap sub-themes to have a successful intervention in that case. Background (sectors, economic income, funding) checked and compared to find differences between themes and sub-themes. |

**Table** **S3**: Main and detailed background information extracted from all reported interventions using the AMR-Intervene framework (3). AMR= antimicrobial resistance; AMS= antimicrobial stewardship; AMU= antimicrobial use; incl = includes; IPC= Infection prevention and control; MDR=multidrug resistance; OH= ‘One Health’; PDR= pandrug resistance; SDR= simple drug resistance; XDR= extensively-drug resistance.
^ = One intervention was implemented locally both in the United States and Brazil and two were implemented globally. *EURO includes: Finland, Germany, Latvia, Luxembourg, Scotland, Sweden and The Netherlands. **AFRO includes: Burundi, Comoro, Djibouti, Eritrea, Ethiopia, Kenya, Rwanda, Seychelles, Somalia, South Sudan, Sudan, Tanzania and Uganda.

| Group (3) | Variables (3) | Categories | | N=77 | Percentage (%) |
| --- | --- | --- | --- | --- | --- |
| Answers quality and description | Sufficient | Good | | **37** | 48% |
|  |  | Succinct | | **27** | 35% |
|  | Insufficient | Vague | | **13** | 17% |
| Social system | Economic scale | High-income countries | | **57** | 75% |
|  |  | Low-middle income countries | | **17** | 22% |
|  |  | Other ^ | | **3** | 3% |
|  | Spatial scale | Pan-Americas Region (PAHO) incl. Canada, United States and Brazil | | **37** | 48% |
|  |  | European Region (EURO) * | | **21** | 27% |
|  |  | South East Asia Region (SEARO) incl. India and Bangladesh | | **12** | 16% |
|  |  | African Region (AFRO) ** | | **4** | 5% |
|  |  | Global | | **2** | 3% |
|  |  | West Pacific Region (WPRO): Japan | | **1** | 1% |
|  | Sector scale | Animal sector | | **37** | 48% |
|  |  | Human sector | | **22** | 29% |
|  |  | Animal and food sectors | | **7** | 9% |
|  |  | Human and animal sectors | | **7** | 9% |
|  |  | Human, animal, food sectors, environment sectors (‘OH’) | | **3** | 4% |
|  |  | Environment or plant sectors | | **2** | 3% |
|  |  | Not specified | | **1** | 1% |
|  | Time scale | Start | 2015-2019 | **43** | 56% |
|  |  |  | 2010-2014 | **15** | 20% |
|  |  |  | Before 2010 | **9** | 12% |
|  |  |  | Not known | **10** | 13% |
|  |  | End | Ongoing | **56** | 73% |
|  |  |  | Delimited | **21** | 27% |
| Governance | Agents responsible | Public sector (government owned) | | **36** | 47% |
|  |  | Public and private sector | | **21** | 27% |
|  |  | Public and academic sector | | **8** | 10% |
|  |  | Private sector (private owned) | | **7** | 9% |
|  |  | Academic sector (university/research/scientific sector) | | **4** | 5% |
|  |  | Public, private and academic | | **1** | 1% |
|  | Level of funding | Public funding | | **37** | 48% |
|  |  | Public and private funding | | **12** | 16% |
|  |  | Private funding | | **5** | 7% |
|  |  | Without funding | | **21** | 27% |
|  |  | Not known | | **2** | 3% |
| Trigger / goals | Trigger of the intervention | Pressure on AMR (high AMU) | | **27** | 35% |
|  |  | State of AMR (increase of AMR) | | **17** | 22% |
|  |  | Pressure and state of AMR (high AMU, increase of AMR) | | **9** | 12% |
|  |  | Drivers of AMR | | **8** | 10% |
|  |  | Impacts of AMR | | **7** | 9% |
|  |  | Pressure and/or state of AMR and impacts of AMR | | **5** | 7% |
|  |  | Not known | | **4** | 5% |
|  | Trigger type | Reactive | | **71** | 92% |
|  |  | Preventive | | **6** | 8% |
|  | Main goal towards intervention | Improve an action | | **42** | 55% |
|  |  | Initiate an action | | **31** | 40% |
|  |  | Maintain an action | | **4** | 5% |
|  | Main strategy | Conservation (reduce/improve AMU) | | **20** | 26% |
|  |  | Conservation and surveillance and/or other | | **17** | 22% |
|  |  | Surveillance | | **12** | 16% |
|  |  | Conservation and containment (reduction of AMR spread) or IPC | | **12** | 16% |
|  |  | Conservation or surveillance and other | | **10** | 13% |
|  |  | Other | | **6** | 8% |
|  | System intervention | Low leverage point | | **76** | 99% |
|  |  | High leverage point | | **1** | 1% |
|  | Level of implementation | National | | **38** | 49% |
|  |  | Sub-national or Regional | | **27** | 35% |
|  |  | Inter-Regional (different countries in the same area) | | **6** | 8% |
|  |  | Local | | **4** | 5% |
|  |  | International (Global) | | **2** | 3% |
| Bio-ecological scale | Type of microorganism | Bacteria | | **41** | 53% |
|  |  | No specific | | **33** | 43% |
|  |  | Bacteria and Fungi | | **3** | 4% |
|  | Level of resistance | Unknown / Not applicable | | **44** | 57% |
|  |  | MDR | | **25** | 32% |
|  |  | XDR | | **4** | 5% |
|  |  | PDR | | **2** | 3% |
|  |  | SDR | | **2** | 3% |
| Assessment | Assessment of the intervention | In progress | | **39** | 51% |
|  |  | Not-evaluated | | **21** | 27% |
|  |  | Evaluated | | **17** | 22% |
|  | Subjective evaluation | Positive | | **72** | 94% |
|  |  | Neutral/Not sure | | **3** | 4% |
|  |  | No | | **2** | 3% |
|  | Unintended outcomes | Not reported | | **76** | 99% |
|  |  | Reported | | **1** | 1% |

**Table S4:** List of all themes and sub-themes. Total number of interventions that reported each of the thirty-two sub-themes, number of interventions in high-income countries (HICs) and number of interventions in low-middle income countries (LMICs). P-value of the Fisher exact test for each theme comparing sub-theme frequencies in HICs and LMICs, comparing sub-theme frequencies of success factors reports and comparing frequencies of obstacles reports. AMU = Antimicrobial use.

| **Theme** | **Sub-theme** | **Total** | **HICs** | **LMICs** | **p-value** | **p-value**  **SF** | **p-value O** |
| --- | --- | --- | --- | --- | --- | --- | --- |
| Behaviour | Collaboration and coordination | 23 | 15 | 6 | 0.96 | 0.86 | 0.93 |
| Behaviour | Commitment and engagement | 19 | 13 | 4 |  |  |  |
| Behaviour | Trust and support | 13 | 10 | 2 |  |  |  |
| Behaviour | Promote, reinforce or award correct behaviour | 9 | 8 | 1 |  |  |  |
| Behaviour | Frustration | 6 | 5 | 1 |  |  |  |
| Behaviour | Communication | 5 | 4 | 1 |  |  |  |
| Behaviour | Flexibility and adaptability | 3 | 3 | 0 |  |  |  |
| Capacity and resources | Personnel | 21 | 16 | 4 | 0.83 | 0.60 | 0.52 |
| Capacity and resources | Funding and finances | 18 | 13 | 5 |  |  |  |
| Capacity and resources | Premises and technology | 13 | 8 | 3 |  |  |  |
| Planning | Implementation | 28 | 19 | 8 | 0.64 | 0.70 | 0.19 |
| Planning | Assessment | 19 | 14 | 5 |  |  |  |
| Planning | Design | 16 | 14 | 2 |  |  |  |
| Information | Awareness | 19 | 14 | 5 | 0.70 | 0.97 | 0.22 |
| Information | Data availability | 12 | 9 | 1 |  |  |  |
| Information | Education | 10 | 8 | 1 |  |  |  |
| Information | Regulations, guides and previous experience or consultancy | 10 | 6 | 3 |  |  |  |
| Information | Outcomes of interventions | 8 | 6 | 2 |  |  |  |
| Intervention | Mandatory enforcement | 19 | 18 | 1 | 0.76 | 0.99 | 0.60 |
| Intervention | Multiple profiles | 19 | 16 | 2 |  |  |  |
| Intervention | Affordable | 5 | 4 | 0 |  |  |  |
| Intervention | Preventive | 3 | 3 | 0 |  |  |  |
| Institution | Management | 21 | 19 | 1 | 0.09 | 0.59 | 0.14 |
| Institution | Governance | 19 | 14 | 5 |  |  |  |
| AMU | Access | 10 | 9 | 1 | 1 | 1 | 1 |
| AMU | Reduction | 9 | 8 | 1 |  |  |  |
| AMU | Improvement | 8 | 6 | 1 |  |  |  |
| AMU | Financial implications | 3 | 3 | 0 |  |  |  |
| Infection Control | Infection or AMR control | 14 | 12 | 2 | 0.08 | 0.35 | 1 |
| Infection Control | Surveillance, epidemiology and preventive screening | 11 | 5 | 5 |  |  |  |
| Research, innovation and novelty | New therapies and alternatives to antimicrobials | 4 | 3 | 1 | 1 | 1 | 0.33 |
| Research, innovation and novelty | Investment in novelty and research | 3 | 3 | 0 |  |  |  |

**Table S5:** Themes (by order in Table 2) and their respective sub-themes reported as success/obstacles of interventions resulting from this thematic analysis. AMR= antimicrobial resistance, AMU= Antimicrobial use; Ob= obstacle; Sf= success factor; T.N. = total number of interventions reporting that subtheme. Note that nine sub-themes were only reported as success factor: (i) communication, (ii) flexibility and adaptability, (iii) education, (iv) outcomes of the intervention,(v) preventive character, (vi) reduction in AMU, (vii) improvement in AMU and (viii) financial implications, and (ix) surveillance, epidemiology and preventive screening. Note that only one sub-theme belonging to the behaviour theme was reported just as an obstacle to intervention success and it was the frustration sub-theme.

| Theme | Sub-theme | T.N. in 77 | Partial contri-bution | | HICs  T.N. in 57 | HICs  Partial contri-bution | | LMICs T.N. in 17 | LMICs  Partial contri-bution | | Meaning and assumptions | Implications | Quotes |
| --- | --- | --- | --- | --- | --- | --- | --- | --- | --- | --- | --- | --- | --- |
|  |  |  | Sf | Ob |  | Sf | Ob |  | Sf | Ob |  |  |  |
| Behaviour | Collaboration and coordination | 23 | 18 | 6 | 15 | 13 | 3 | 6 | 4 | 2 | Actors need to collaborate and/or coordinate themselves to enhance likelihood of success. Collaboration and coordination lead to effective communication and implementation. On the contrary, reluctance to participate, lack or difficult collaboration, disagreements and lack of coordination with mixed tasks can jeopardize the intervention. | A collaborative and coordinative behaviour is crucial to involve individuals into AMR, and to engage them clearly in the specific tasks they need to do with motivation. | “Open collaboration between all entities”// “cooperation with food producers and cattle farms…”// “…and collaborative effort between industry and government”// “A key factor is the good collaboration between national and local […] groups…”// “Challenges included attaining industry buy-in and collaboration, considering that each livestock industry has its unique considerations,…” |
| Behaviour | Commitment and engagement | 19 | 16 | 4 | 13 | 11 | 3 | 4 | 3 | 1 | Commitment, engagement and implication of actors is crucial to conduct things well and in a positive way as people welcome the campaign and have willingness to take actions including voluntary actions. | Actors that are committed believe that what they are doing help in tackling AMR and are more aware and willing to (voluntarily) participate and take action. | “The swine stakeholder (…) voluntarily committed to reduce antimicrobial use by 20%” // “Implication of the stakeholders, communication with the staff” // “The initiative was also successful due to outreach and engagement with farm and veterinary communities …” // “Challenges included engagement and negotiation with industry around costs of antimicrobial stewardship,…” |
| Behaviour | Trust and support | 13 | 4 | 9 | 10 | 3 | 7 | 2 | 0 | 2 | Trust, support and confidence between actors are essential for acceptance of interventions. On the contrary, prejudice, scepticism, concern, fear (of consequences) caused by interventions and mistrust or competition of actors hinders performance of interventions. | Positive attitudes such as trust, confidence and support between actors are essential to positively believe in what has been or is going to be done. They enhance implementation and maintenance of interventions. | “…total faith in government and Public Health Institutions.”// “National and European authorities support”// “Concern about how to manage disease without antimicrobial prophylaxis”// “Misunderstood competition between healthcare providers are initially a hurdle…” |
| Behaviour | Promote, reinforce or award correct behaviour | 9 | 6 | 5 | 8 | 6 | 4 | 1 | 0 | 1 | New routines or ideas must be promoted during long periods, reinforced and/or awarded to ensure continuity. Habituation needs time and going back to old routines due to inertia or resistance to change is usual. | Training and guidance are essential to make changes in the long term. Follow-up and regular feedback maintain motivation. Sustained efforts and interventions plus constant reminders are needed to avoid old habits. | “Repetition of the message: repeat, repeat, repeat. Otherwise, dilution over time due to new personnel, not remembering or forgetting actions. Repeat, repeat and repeat as effective solution.”// “Money reward if they agree: bonus. (…) lobbing indirect work (…) but rewarding the ones that actually are doing the right thing.” |
| Behaviour | Frustration | 6 | 0 | 6 | 5 | 0 | 5 | 1 | 0 | 1 | Feeling of being upset, sad or annoyed by the interventions, their actors or the system in which are implemented. Frustration has been consequence of discrimination between health professionals, attitudes not prioritizing AMR or injustice due to more permissiveness depending on the location. | This attitude leads to disbelief or hopeless behaviours that result in inability to change, implement, or achieve the goals of interventions. | “That many of the agencies have so many other important subjects to focus on.” // “Growers in Canada were interested […] because U[nited]S[tates] growers had these "tools" available to them for a variety of crop diseases.”// “…but the outcome is increased burden on the already stretched healthcare infrastructure; like even for all lab related reports,  now there needs to be a doctor present as a person to sign. How is that going to help the patients in real need. In a developing country like India where there is already shortage of doctors, this bill has shifted the focus from patient care to doctors as the sole signatories for the lab reports. That has affected the microbiologists and molecular biologists who are into AMR…” |
| Behaviour | Communication | 5 | 5 | 0 | 4 | 4 | 0 | 1 | 1 | 0 | Transmission of simple clear ideas, done in the language actors speak and/or with images and visual messages help towards the design, needs and implementation of interventions. | A behaviour that influences positively how the message is transmitted and understood and attitudes that enhance communication impact positively intervention outcomes. | “Effective communication solves lot of issues in AMR.” // “…national agencies work together with the same problem and message to the public”// “All conferences are in French (which is the official lang[u]age…”// “A message simple, strong and common to the partners…” |
| Behaviour | Flexibility and adaptability | 3 | 3 | 0 | 3 | 3 | 0 | 0 | 0 | 0 | Flexible attitudes and behaviours to adapt interventions depending on the system and on specific and detailed context. | Capacity of adequate each intervention to the context in which its taking place is important to have clear, personalized, realistic and tailored goals. | “…free veterinary visits to swine producers. One of the goals of the veterinary visits is to ensure the judicious use of antibiotics and to put in place a personalized plan…” // “…discussion with our producers, as well as with our hatchery partners as there are provincial and regional production differences, so a national requirement has to be flexible enough to take these differences into account..” |
| Capacity and resources | Personnel | 21 | 1 | 20 | 16 | 0 | 16 | 4 | 1 | 3 | Personnel and/or trained personnel working on the intervention. On the contrary, lack of them, or personnel with heavy overload schedules without sufficient time or personnel unable to assist, for example in remote or rural areas hinder intervention outcomes. | Personnel accessible, dedicated and with enough time to carry out the intervention or only working on the intervention is needed to ensure likelihood of success and that the actions expected from the interventions are met. | “availability of human resources” // “Personnel exclusively dedicated to that” // “availability of time, work initiated in the summer” // “Lack of experts and public diagnostic facilities for AMR-prevention”// “The availability to inf[ectious] dis[eases] specialists and the financing of the time it takes to do the rounds” |
| Capacity and resources | Funding and finances | 18 | 2 | 16 | 13 | 2 | 11 | 5 | 0 | 5 | Enough budget and funding to carry out all aspects needed for interventions overtime. Funding for resources, techniques or personnel, but also for teaching and training the main actors responsible of the intervention. | Good budgets are key as costs can be very expensive for implementing interventions. Without enough budget many interventions are not going forward, are partially applied, interrupted or side cost effects are assumed by others (with negative effects). | “Founder donor agency go through a complicated process which causes interruption of funding.” // “There was no dedicated budget for this campaign. Communication strategy was based primarily on the information relay and the ability of each organization to pay for the printing of the tools and their distribution.”// “Financial resources and education” // “Funding” |
| Capacity and resources | Premises and technology | 13 | 4 | 9 | 8 | 2 | 6 | 3 | 1 | 2 | Adequate and accessible equipment, tools, software and lab and diagnostic facilities to conduct the intervention. Moreover, capacity of adapt and share these resources. | Accessible tools and equipment, high tech labs, regional and national diagnostic facilities, free and/or online shared software among actors improves the performance of interventions. | “Lack of experts and public diagnostic facilities for AMR-prevention”// “IT related problems, connection between the national system and the patients records in the regions.”// “Laboratory capacity for the identification of MDRO”// “Laboratory capacity to undertake surveillance activity” |
| Planning | Implementation | 28 | 20 | 11 | 19 | 13 | 9 | 8 | 6 | 2 | Implementation planning needs to be very well detailed, easy to apply and considering flexibility of contexts and to be tailored to them. It must also have consultation or guidance for actors during implementation to clarify actions and objectives of the intervention. When lacking, often implies insecurity towards the intervention and actors can go back to old habits especially if the implementation process is ling or requires a certain amount of time. | Strong implementation consider small scale contexts (e.g. regional) even though interventions can be implemented at bigger scales (e.g. national). Guidance enhances positive outcomes, even though if the implementation is long, process as they can rely on experts or other professional’s criteria when doubts arise. It promotes self-esteem and motivation of executors due to continuous knowledge, feedback and follow-ups. | “…there are provincial and regional production differences, so a national requirement has to be flexible enough to take these differences into account…”// “It takes a lot of time to implement a program that is supposed to reach all nurses in all hospitals”// “resistance to change - this change took over 10 years to implement!”// “But we also understood that it would take time to implement in all hospitals…Step by step we learn more with national and regional workshops to share experience”// “[Implementation] Guidance from WHO, OIE and FAO” // “Support from WHO & AGISAR documents” |
| Planning | Assessment | 19 | 18 | 1 | 14 | 14 | 0 | 5 | 4 | 1 | After implementation, checking, analysing or measuring outcomes of the actions applied can help to elucidate the usefulness of the intervention or its possible gaps, otherwise, the usefulness is not assessed and, therefore, unknown. | Results from assessment can help to maintain motivation if there are positive outcomes and to identify new goals and opportunities to improve outcomes or to promote actions impacting AMR. | “Dialogue based on the figures for each unit and they can see differences between units and colleagues.” // “[Assessment with] quantifiable objective”// “Clinical microbiology laboratories are typically required to provide annual [data]… to providers to help guide empiric antimicrobial therapy.”// “to obtain enough microbiological data to follow temporal trends of antimicrobial resistance.” // “Impact of awareness creation needs to be evaluated” |
| Planning | Design | 16 | 12 | 4 | 14 | 10 | 4 | 2 | 2 | 0 | Time to plan and design interventions. Good planning has well defined targets, detailed timeline and it can foresee if training of professionals is needed or if possible complications and where can arise. | Preparedness and time to carefully think on the system interventions are embedded is key for having the desired outcomes. | “This was discussed at length before the intervention was…implemented.” // “… systematic collection, aggregation and analysis of AMR data representing all the geographical areas of the country is being done. Lab capacity in terms of manpower development (through training by ASM members), providing laboratory SOP logistics and equipment has been developed, a software capable of collecting all the lab and epi[demiological] data has been developed to collect both kind of data from different sentinel sites to the center… in real time.”// “short timeline to create and deliver a national awareness campaign”// “Cost is always an obstacle as interventions typically add cost to operations; this is typically discussed before the intervention is finalized and implemented.” |
| Information | Awareness | 19 | 14 | 7 | 14 | 11 | 3 | 5 | 3 | 4 | Knowledge about AMR and people aware of the problem of untreatable infections enhance positive outcomes and thorough follow of therapy. Ignorance of the problem may lead to pressure for antibiotic prescribing and public opposition. | Society may behave differently following and finishing prescribed antimicrobial treatments. Prescribers less pressured to prescribe treatments to please patients or farmers. Citizenship is engaged to preserve antimicrobial effectiveness. | “Consistent awareness creation, commitment” // “Public awareness by showing who received certificates. Certificates were handed over by publicly important personalities, such as health ministers or regional governors.  - Public awareness through modern media (TV, radio)”// “This Annual Conference recalls the importance of the issue of antimicrobial resistance.” // “industry-wide initiative that expanded past our sector, and was accompanied by regular communication to farmers to increase awareness” |
| Information | Data availability | 12 | 8 | 4 | 9 | 5 | 4 | 1 | 1 | 0 | Data collection and provision is systematic, standardized, available and shared. On the contrary, data collected or provided from different sites, with heterogeneous criteria or not shared hinders availability of knowledge | Data of interventions that are shared, with standard reporting can clarify the exact situation of the epidemiological state, it can be used by different settings or sectors and it can clarify and/or quantify assessments. | “The number of countries participating in the data collection has been increasing, and countries report an increased level of quantitative detail in the data they provide.” // “information collection and collaboration” // “Having provincial laboratories…to obtain enough microbiological data to follow temporal trends of antimicrobial resistance.”// “Resources for data collection and reporting” |
| Information | Education | 10 | 10 | 0 | 8 | 8 | 0 | 1 | 1 | 0 | Deep and detailed knowledge or education increases system capacity to carry out the intervention. Online courses and free tools can be useful to reach all. | Contribution and expansion of skills and knowledge to new staff or new performers with personal feedback. Impede waning of the intervention. | “The intervention was developed from high-level clinical cases” // “It gives important data for feed-back to the doctors. Diagnosis linked prescription”// “The challenge encourages medical students, trainees, physicians, and others to collect or write cases to be used in medical education and shared in an online resource that is free to access.” |
| Information | Regulations, guides and previous experience or consultancy | 10 | 4 | 7 | 6 | 3 | 3 | 3 | 1 | 3 | Clear and the same regulations and/or guides to follow help to better implement the intervention. Previous experiences of actors or consultants also help to know what needs to be enhanced. If there are different regulations or guides to follow (e.g. between countries) or they do not exist implementation is challenged. | If regulations or guides clash, there can be disagreements in which ones to apply. If there is a lack of them, information about the context of the implementation may be missing. Plus, if there is lack of reference models or expertise of actors, implementation of the intervention could be weak. | “1. Lack of regulatory framework;…” // “Funding, regulatory framework” // “Lack of a reference model on Veterinary surveillance” // “Expertise […] for testing AMR…” // “Support from WHO & AGISAR documents” // “The 'borders' between the sectors science, health and business and the border between Netherlands and Germany.” |
| Information | Outcomes of interventions | 8 | 8 | 0 | 6 | 6 | 0 | 2 | 2 | 0 | When interventions are assessed or partially assessed, the outcomes can be useful to know what to transform in the system, what to improve or what to expect from interventions. | Outcomes can be used as a tool for improving, for enhancing positive changes, for updating priority actions or for gazetting guides. | “It gives important data for feed-back to the doctors. Diagnosis linked prescription.”// “Its helped a lot that […] publication from 2015 in Southern Sweden proving that antibiotic rounds could reduce total a[nti]b[iotic] consumption with 27%.”// “they were provided the results on occurrence of antimicrobial resistance within their herds or food produce and, as outcome of the project, insight on possible measures to implement.” |
| Intervention | Mandatory enforcement | 19 | 16 | 4 | 18 | 16 | 3 | 1 | 0 | 1 | When interventions are mandatory, actors need to implement and comply with what is mandate, independently of what they think or their preferences. | Intervention has to be implemented by main actors, they do not necessarily need to be interested (so it is not siloed to the ones who already care like voluntary interventions). | “Strict government regulation and requirement to reduce antimicrobial use in food animals at the national level”// “It was a mandatory reduction in use”// “it was successful because it was mandated - farmers had to comply”// “Regulatory authority saw it important to make sure that the legislation was obeyed.” |
| Intervention | Multiple profiles | 19 | 17 | 2 | 16 | 16 | 0 | 2 | 1 | 1 | Interventions whose responsible actors are from different sectors (Multisector, One Health), disciplines (multidisciplinary/ transdisciplinary) or have different roles in the same action or complemented actions (multifaceted). Intervention is composed or carried out by different actors in sectors, settings, disciplines or professional background. | Different professionals, sectors and disciplines help to understand and detail better the variety and complexity of AMR and have more insights on how to tackle this challenge. Joined efforts from different backgrounds and perspectives may have bigger impacts and redundancy. | “…collaborative effort between industry and government” // “The cross border aspect, transdisciplinary, regional network formin[g]a common goal.” // “multi-disciplinary team from industry, academia and government” // “inability to accept multidisciplinary or varied thought processes”// “Multisector approach” // “One Health approach” |
| Intervention | Affordable | 5 | 4 | 1 | 4 | 3 | 1 | 0 | 0 | 0 | When interventions are affordable, with low investment and there is no need of big resources are more prone to be implemented. | Costly or expensive interventions are difficult to implement as finding the budget for them can be difficult and they may be at risk of not being implemented. | “Having provincial laboratories of animal health that offer necropsy and laboratory analyses at affordable prices allows MAPAQ to obtain enough microbiological data to follow temporal trends of antimicrobial resistance.” // “Improved bacterial control without major investment”// |
| Intervention | Preventive | 3 | 3 | 0 | 3 | 3 | 0 | 0 | 0 | 0 | Interventions that have a preventive character, tackling and issue is not there yet, have more preparedness and knowledge of the problem. | Tackling a problem is much more difficult than preventing it or delaying it. | “Precautionary principle was perhaps applied in order to protect efficacy of these new medicines.” // “…antimicrobial cascading allows only those antibiotics that you wish a doctor to use to be released on a patient lab report.” |
| Institution | Management | 21 | 17 | 4 | 19 | 16 | 3 | 1 | 1 | 0 | Execution of interventions suggesting how interventions are going to be done (either designed, implemented or assessed). Management has communication as a key skill to drive and organize all the pieces of the intervention. | Good management foresees how to train, how to coordinate, or how to enhance collaboration of actors. This empowers and increases information available in the system, plus it increases knowledge and self-esteem. | “That so many Swedish national agencies work together with the same problem and message to the public.” // “Regional training activities” // “Implication of the stakeholders, communication with the staff” // “open collaboration between all entities”// “Educational afternoons for them, updates and workshops. Practical information on how to treat infections.” |
| Institution | Governance | 19 | 15 | 4 | 14 | 13 | 1 | 5 | 2 | 3 | Compromise, commitment, engagement, support and clarity towards the intervention, its goals and decisions from the institution suggesting what should be done or being accountable for interventions. | Ensures balanced effort and the broader interests from the institution to maintain or to carry out the intervention. This is done, independently of individualities and personal interests, joining efforts in partnerships and avoiding hierarchy or roles of power. | “Long-term government engagement of stakeholders”// “Good political support” // “Governance of the programme and financial commitment” // there is a need to strength[en] the relation between Academia-Governmental institutions”// “Achieve good multisector collaboration and bureaucratic procedures between institutions from different origins.” |
| AMU | Access | 10 | 6 | 4 | 9 | 6 | 3 | 1 | 0 | 1 | Access to antimicrobials is controlled, monitored and linked to prescriptions. This implies that prescribers need to be also accessible and that has been seen as a hurdle in remote or rural areas. | Antimicrobials are more difficult to buy as you need prescription for them. Over-the-counter use is forbidden and you have more control about the consumption of antimicrobials. | “Concern on the access to the drug products and veterinarians.”// “this represented a challenge with timely access for some farmers and their treating veterinarians who may not be able to arrange timely dispensing for reasons of distance, storage at the veterinary clinic or inconvenience.”//” all systemic antimicrobials were given a prescription-only status in the legislation.” |
| AMU | Reduction | 9 | 9 | 0 | 8 | 8 | 0 | 1 | 1 | 0 | AMU has been the major driver of AMR. Reducing AMU is key to tackle AMR and to preserve effectiveness of antimicrobials. | AMU reduction implies less selection pressure towards microorganisms. | “Awareness of veterinarians about critically important antimicrobials and their prudent use increased and the usage of these antimicrobials decreased markedly.” // “The initiative has a measurable target (20% reduction) in use to a period for which we already have measures.” // “It was a mandatory reduction in use” |
| AMU | Improvement | 8 | 8 | 0 | 6 | 6 | 0 | 1 | 1 | 0 | AMU has been the major driver of AMR. Improving AMU with judicious use is key to tackle AMR and to preserve effectiveness of antimicrobials, especially of key antimicrobials with broad spectrum. | AMU improvement can imply reduction in use but not always. It can also imply a shift towards narrow-spectrum antimicrobials to preserve key antimicrobials, selecting and reported these and escalating to broad ones if needed. | “… antimicrobial cascading allows only those antibiotics that you wish a doctor to use to be released on a patient lab report. In this way, we can promote use of narrow-spectrum antimicrobials…” // “Selective reporting of drugs too for example we don't report quinolones for Staphylococcus aureus as it might select out MRSA” |
| AMU | Financial implications | 3 | 3 | 0 | 3 | 3 | 0 | 0 | 0 | 0 | Rewards if actors do the intervention correctly or no financial benefits when prescribing antimicrobials. | Prescribing or selling antimicrobials only if needed as there is no personal economic interest behind or rewards for doing the right thing and comply with the intervention. | “some money reward if they agree on that: bonus. Result that they have been lobbing indirect work instead of telling what to do but rewarding the ones that actually are doing the right thing.” // “As veterinarians have not been able to make profit by selling antimicrobials, they have had no financial interest in using them more than absolutely necessary.” |
| Infection Control | Infection or AMR control | 14 | 10 | 6 | 12 | 8 | 6 | 2 | 2 | 0 | Prevention or reduction of infections or AMR microorganisms using biosecurity, safety programs or sanitation instead of using antimicrobials including meta- or prophylactically. | Reducing infections means less need for antimicrobials. When implementing biosecurity programs that withdraw antimicrobials, if not well done infection incidence can increase. | “Workforce in infection prevention”//“high mortality rates so better [sanitation] management needed to be 'taught'” // “disease issues from reduced antimicrobial use” // “over 75% reduction in food borne Significantly lower loading rates of A[ntimicrobial]R[esistance]G[ene]s compared to anaerobically digested sludge  infections through processed foods”// “” |
| Infection Control | Surveillance, epidemiology and preventive screening | 11 | 11 | 0 | 5 | 5 | 0 | 5 | 5 | 0 | Information about the current epidemiological situation with continued surveillance, antimicrobial susceptibility testing and, in some settings, preventive screening. | These tools can help to better manage AMR, useful detailed data to know what is more prevalent including species and subspecies data. | “Understand epidemiology at a subspecies level, as species level does not allow to understand real epidemiology” // “- Implementation of preventive screening”// “Antibiogram development (i.e. antimicrobial resistance surveillance in human pathogens) has been common practice in clinical microbiology laboratories for many years.”// “…obtain enough microbiological data to follow temporal trends of antimicrobial resistance.” |
| Research, innovation and novelty | New therapies and alternatives to antimicrobials | 4 | 4 | 1 | 3 | 3 | 0 | 1 | 1 | 1 | There is a need for new antimicrobials or therapies that use alternatives to antimicrobials to treat infections. | New treatments can help to treat infections with few therapeutic options and avoid mortality and morbidity. | “anti-biofilm compound will help to reduce the use of antibiotics”// “It works through a principal of "microflora management" instead of using antimicrobials towards which microbes will inevitably develop resistance.”// “screening of potential drug candidates and drug target selection is a bit time consuming process.” |
| Research, innovation and novelty | Investment in novelty and research | 3 | 1 | 2 | 3 | 1 | 2 | 0 | 0 | 0 | Research, innovation and novelty usually need investment without knowing the final outcomes of the process. | Research is often expensive, not always you can foresee the real cost and not always ends up with rewards which implies that many institutions do not prioritize them. | “Increasing R&D budgets for reaching of milestones”// “funding of peptide drug discoveries is very challenging”// “More research is currently funded and underway” |

**References:**

1. O’Brien BC, Harris IB, Beckman TJ, Reed DA, Cook DA. Standards for reporting qualitative research: A synthesis of recommendations. *Acad Med* (2014) 89:1245–1251. doi: 10.1097/ACM.0000000000000388

2. Braun V, Clarke V. Using thematic analysis in psychology. *Qual Res Psychol* (2006) 3:77–101. doi: 10.1191/1478088706qp063oa

3. Léger A, Lambraki I, Graells T, Cousins M, Henriksson PJG, Harbarth S, Carson C, Majowicz S, Troell M, Parmley EJ, et al. AMR-Intervene: a social–ecological framework to capture the diversity of actions to tackle antimicrobial resistance from a One Health perspective. *J Antimicrob Chemother* (2021) 76:1–21. doi: 10.1093/jac/dkaa394
